# Bicyclic Engineered Sortase A Performs Transpeptidation under Denaturing Conditions

**DOI:** 10.1021/acs.bioconjchem.3c00151

**Published:** 2023-05-29

**Authors:** Sebastian Kiehstaller, George H. Hutchins, Alessia Amore, Alan Gerber, Mohamed Ibrahim, Sven Hennig, Saskia Neubacher, Tom N. Grossmann

**Affiliations:** † Incircular BV, De Boelelaan 1108, 1081 HZ Amsterdam, The Netherlands; ‡ Department of Chemistry & Pharmaceutical Sciences, Vrije Universiteit Amsterdam, De Boelelaan 1083, 1081 HV, Amsterdam, The Netherlands; § Amsterdam Institute of Molecular and Life Sciences, Vrije Universiteit Amsterdam, De Boelelaan 1083, 1081 HV, Amsterdam, The Netherlands

## Abstract

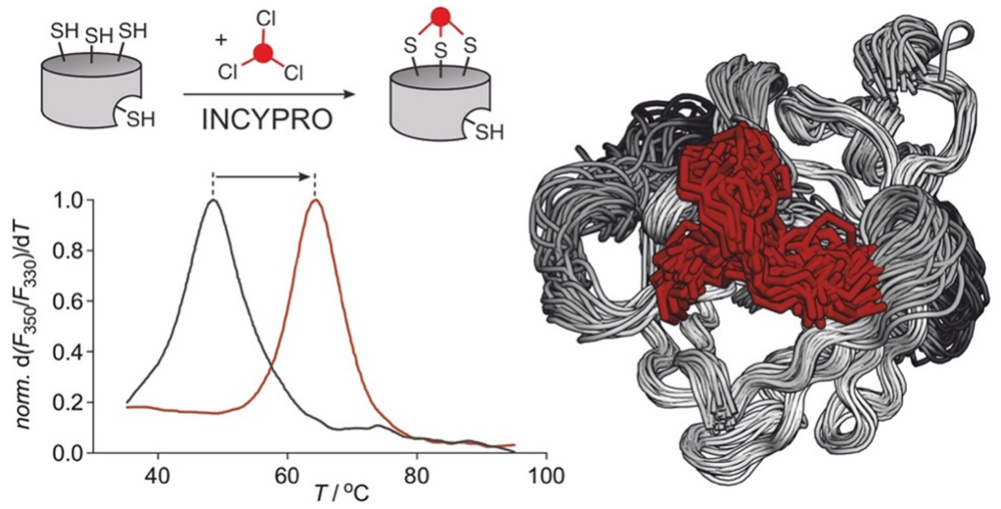

Enzymes are of central importance to many biotechnological and biomedical applications. However, for many potential applications, the required conditions impede enzyme folding and therefore function. The enzyme Sortase A is a transpeptidase that is widely used to perform bioconjugation reactions with peptides and proteins. Thermal and chemical stress impairs Sortase A activity and prevents its application under harsh conditions, thereby limiting the scope for bioconjugation reactions. Here, we report the stabilization of a previously reported, activity-enhanced Sortase A, which suffered from particularly low thermal stability, using the *in situ* cyclization of proteins (INCYPRO) approach. After introduction of three spatially aligned solvent-exposed cysteines, a triselectrophilic cross-linker was attached. The resulting bicyclic INCYPRO Sortase A demonstrated activity both at elevated temperature and in the presence of chemical denaturants, conditions under which both wild-type Sortase A and the activity-enhanced version are inactive.

## Introduction

The efficiency and selectivity of enzymes are central to biocatalytic, biotechnological, and diagnostic applications.^1,2^ Conditions relevant to these applications, however, often involve thermal or chemical stress, which can severely impact enzyme activity.^3^ Increasing the stability of enzymes without impeding their catalytic activity is therefore of central importance to facilitate broad enzyme applicability.^4^ The stabilization of enzymes can be achieved, e.g., via consensus-based mutagenesis, computational design, the introduction of unnatural hydrophobic amino acids, or directed evolution.^5−9^ These strategies usually require iterative optimization cycles and result in the introduction of various amino acid variations. In natural proteins, tertiary structures are frequently stabilized via intradomain disulfide bridges between two spatially aligned cysteines.^10^ Using so-called disulfide engineering, such bridges have been newly introduced to increase protein stability.^10,11^ Approaches that use nonproteinogenic bridging scaffolds have also been reported providing access to a broader range of cross-linking sites within a given protein.^12−19^ If appropriately positioned they can stabilize protein structures, however, often multiple cross-linkers have to be installed to achieve meaningful stabilization.^14,15,19^

The transpeptidase Sortase A (SrtA) is an enzyme that is widely used for protein modification involving diverse applications such as protein labeling, bioconjugation, and immobilization.^20−25^ For various applications, the sensitivity of SrtA to thermal and chemical stress is a limiting factor.^26,27^ We have recently reported the *in situ* cyclization of proteins (INCYPRO), an approach that facilitates the construction of bicyclic proteins in a single design step.^19,28^ INCYPRO is structure-based and uses C3 symmetric tris-electrophiles to covalently link three cysteine residues that have been introduced in spatial proximity. Previously, we have described an INCYPRO-stabilized SrtA that was derived from wild-type *Staphylococcus aureus* SrtA.^19^ While the stabilization effect was considerable (Δ*T*_m_ = 12 °C), the generally low activity of *S. aureus* SrtA in non-membrane-templated transpeptidation reactions limits its usefulness. It would be desirable to obtain a more active stabilized version of SrtA. Herein, we use an activity-enhanced SrtA^29,30^ as a starting point to design a stable, efficient enzyme via INCYPRO cross-linking. Obtained bicyclic SrtA **xS11** shows greatly enhanced activity under denaturing conditions.

## Results and Discussion

### INCYPRO Modification of Engineered SrtA

**Figure 1:**
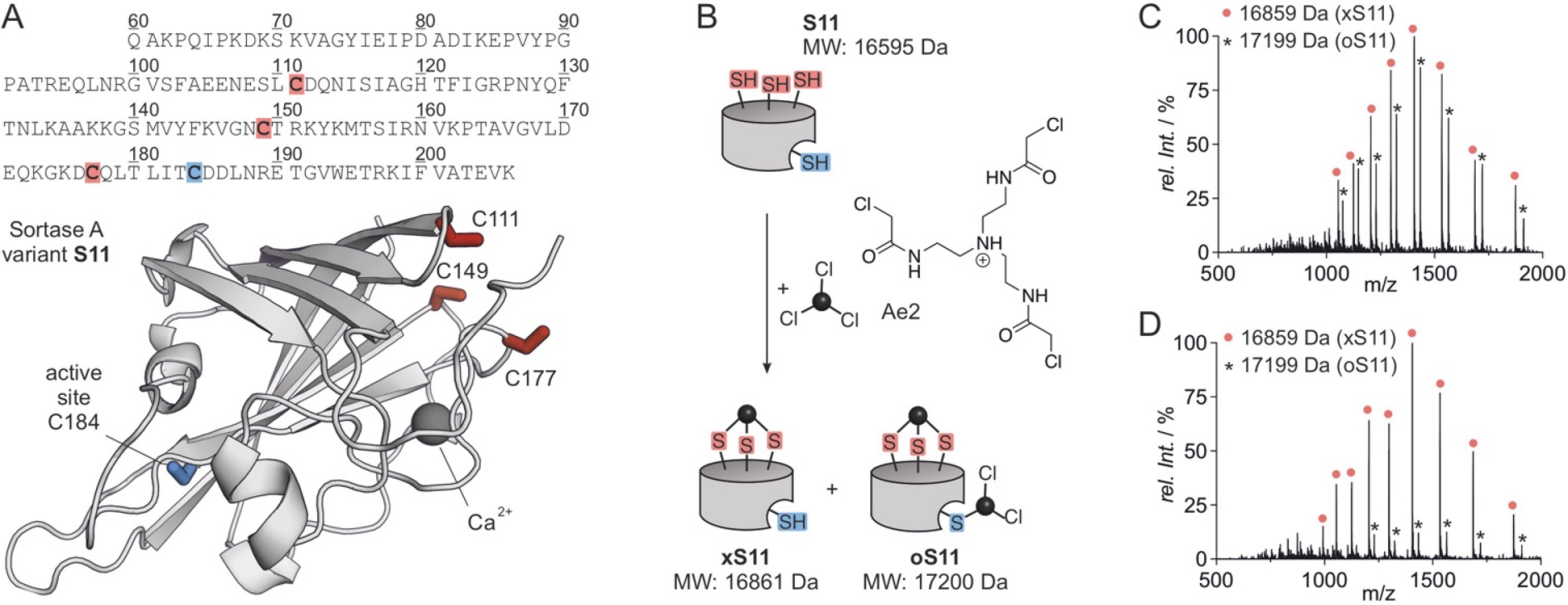
(A) Amino acid sequence of SrtA variant **S11** including homology model (atomic coordinates available as Supporting Information). (B) Reaction of **S11** with Ae2 resulting in desired cross-linked product **xS11** and overalkylated species **oS11**. Calculated molecular weights are stated. (C) MS of product mixture resulting from reaction of **S11** with Ae2 in the presence of 5 mM CaCl_2_. Deconvoluted found masses are stated (for details see Supplementary Figure S3). (D) MS of reaction of **S11** with Ae2 in the absence of CaCl_2_. Deconvoluted found masses are stated (for details see Supplementary Figure S7).

Several engineered variants of Sortase A with enhanced activity and conjugation efficiency have been reported.^23,29−31^ Herein, we use a version reported as the 5M/D124G/Y187L/E189R variant (**8M**), containing a total of eight mutations relative to wild-type SrtA.^29,30^ We applied the INCYPRO stabilization approach to the **8M** variant aiming to produce a bicyclic engineered SrtA with increased thermal stability. Three cysteines were introduced at positions (D111C, E149C, and K177C; Figure 1A, red) previously reported for the INCYPRO stabilization of wild-type SrtA,^19^ resulting in variant **S11** with a total of 11 mutations relative to the wt SrtA (Supplementary Figure S1).

The His_6_-tagged variant **S11** was heterologously expressed from *Escherichia coli* and after tag-cleavage and purification obtained in high purity (Supplementary Figure S2). **S11** was then subjected to tris-electrophile Ae2 (Figure 1B) bearing three cysteine-reactive chloroacetamide moieties.^28,32,33^ Initial cross-linking attempts resulted in the formation of two species as detected by mass spectrometry (Figure 1C, Supplementary Figure S3). One was the desired product **xS11** which had reacted with one equivalent of Ae2; the other one was a side product (**oS11**) with a mass corresponding to product **xS11** plus an additional Ae2 molecule that would still bear two intact chloroacetamide groups (Figure 1B). We accredited this species to the alkylation of the endogenous active site cysteine (C184) representing the only other cysteine in **S11**. In line with this observation, the treatment of **8M** and **S11** with iodoacetamide resulted in one and four modified cysteines, respectively (Supplementary Figures S4 and S5). Furthermore, side product **oS11** does not react with iodoacetamide, whereas **xS11** is alkylated by one molecule of iodoacetamide (Supplementary Figure S6).

Interestingly, an overalkylated side product was not observed during cross-linking of the three-cysteine variant of wild-type SrtA,^19^ suggesting that the active site cysteine (C184) in engineered variants **8M** and **S11** exhibits increased activity and/or accessibility. It has been reported that Ca^2+^ ions are required for sortase activity, putatively stabilizing the active conformation of the substrate binding pocket.^34−36^ We therefore suspected that Ca^2+^-depletion from the cross-linking reaction may reduce overalkylation of the active site cysteine. In the absence of Ca^2+^, we then indeed observed strongly reduced formation of the overalkylated side produce **oS11** while not affecting the formation of **xS11** (Figure 1D, Supplementary Figure S7).

### Purification of INCYPRO Cross-Linked xS11

**Figure 2:**
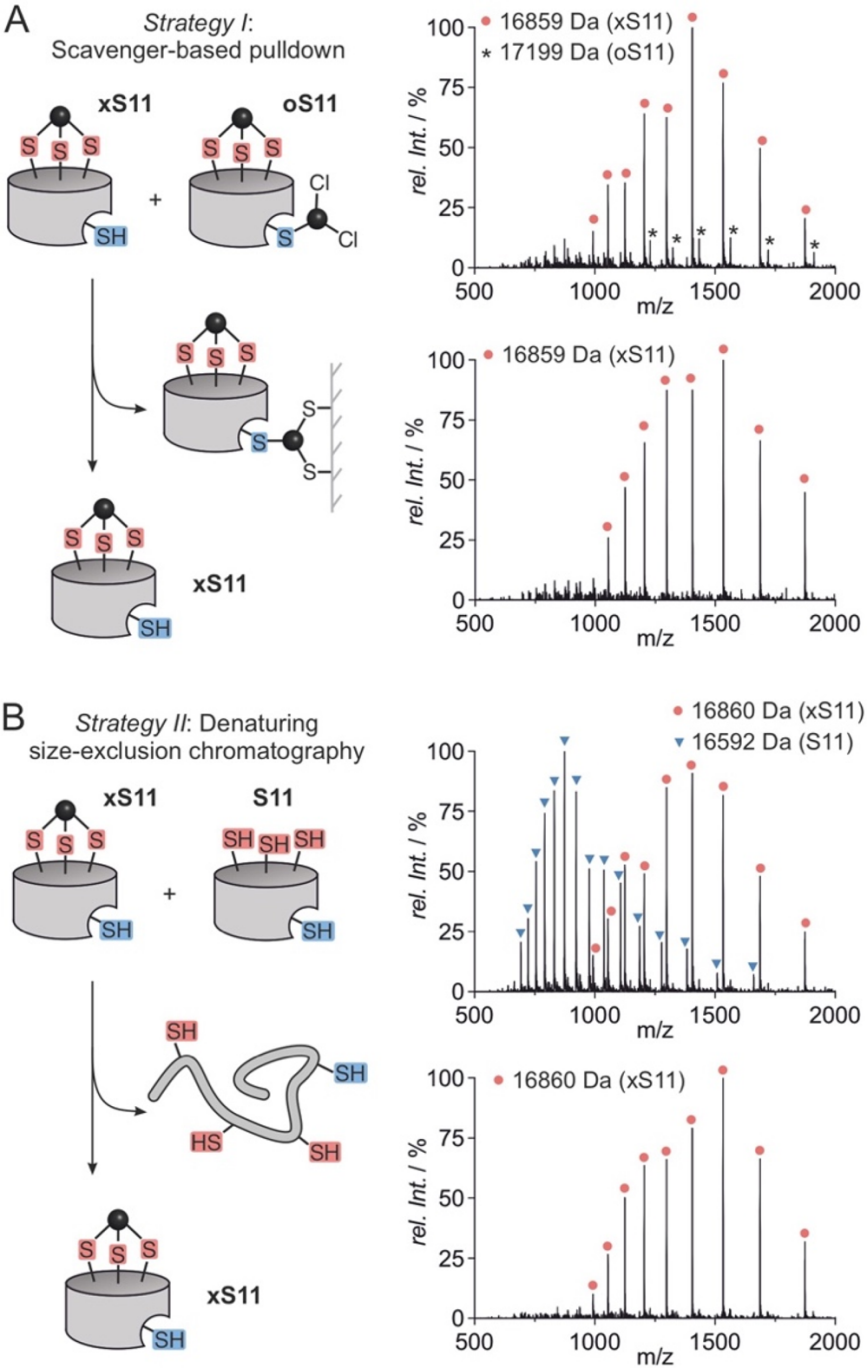
(A) *Strategy I*: The residual chloroacetamide groups on an overalkylated **oS11** are reacted with a biotinylated thiol and removed using streptavidin resin. MS spectra before (top) and after (bottom) purification are shown. Deconvoluted found masses are stated (for details see Supplementary Figures S8 and S9). (B) *Strategy II*: Reaction of **S11** with Ae2 is not allowed to complete, and a mixture of **S11** and **xS11** is subjected to denaturing size exclusion chromatography for separation. MS spectra before (top) and after (bottom) purification are shown. Deconvoluted found masses are stated (for details see Supplementary Figures S10 and S12).

While depletion of Ca^2+^ could reduce formation of the overalkylated product **oS11**, it could not be completely avoided. To further purify **xS11**, we aimed to remove the side product **oS11** from the reaction mixture. For that purpose, the remaining two chloroacetamide moieties of the Ae2 which had reacted with the active site cysteine were exploited for a scavenger-based pulldown (*strategy I*, Figure 2A). The reaction mixture containing both **xS11** and **oS11** was incubated with a biotinylated thiol as a bait (Supplementary Figure S8). Subsequently, biotin-modified **oS11** was immobilized on streptavidin resin enabling its depletion from the solution to provide pure **xS11** (Figure 2A bottom right, Supplementary Figure S9).

As an alternative purification strategy, we aimed to utilize the expected increased stability of **xS11** (compared to **S11**) and its tolerance to denaturants (*strategy II*, Figure 2B). Here, a shorter reaction time and a reduced amount of Ae2 (5- vs 10-fold access) were used to prevent formation of side product **oS11**. This resulted in a mixture of desired product **xS11** and unreacted starting material **S11** (Supplementary Figure S10). In the presence of the denaturant urea (*c* = 2.5 M), **S11** shows a reduced retention volume in size exclusion chromatography suggesting unfolding (Supplementary Figure S11). This change is less pronounced for cross-linked **xS11**, enabling partial separation and isolation of pure **xS11** as confirmed by mass spectrometry (bottom, Figure 2B). After removal of urea by buffer exchange, cross-linked **xS11** retains the expected activity, verifying its functionality after the purification procedure (Supplementary Figure S13).

### xS11 Is Active under Denaturing Conditions

**Figure 3:**
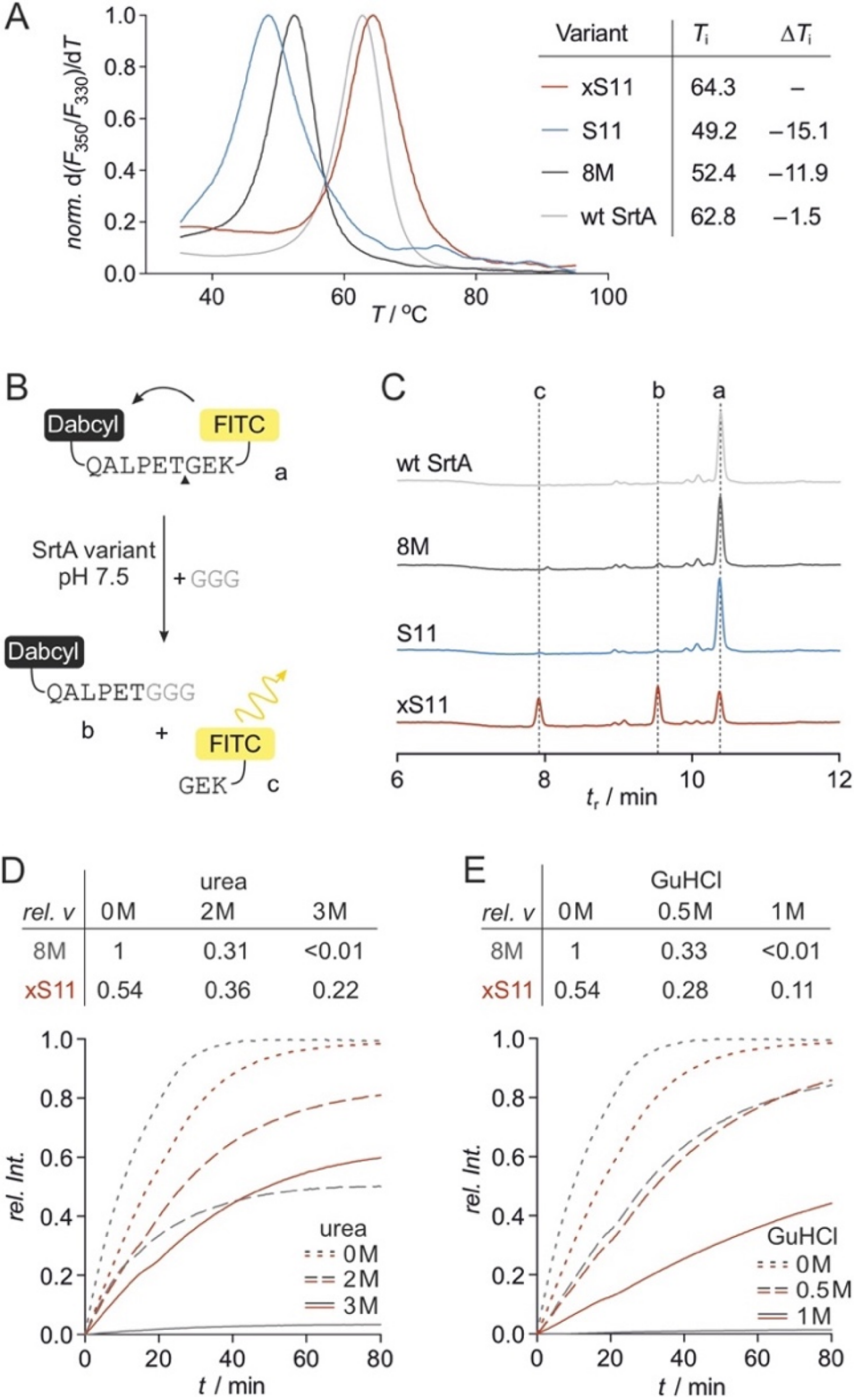
(A) Normalized first derivative of fluorescence ratio (λ = 350 nm/330 nm). Maxima-derived melting temperatures (*T*_i_) are given (for details see Supplementary Figure S16). (B) Principle of transpeptidation activity assay, involving the cleavage of a dual-labeled substrate with fluorophore: fluorescein isothiocyanate (FITC), and quencher: 4-((4-(dimethylamino)phenyl)azo)benzoic acid (Dabcyl). (C) HPLC analysis of transpeptidation reactions performed at 60 °C after 30 min. Signal (a) corresponds to the substrate, while signals (b) and (c) correspond to the products of transpeptidation (for MS spectra see Supplementary Figure S18). (D) Fluorescence intensity measurement of transpeptidation reaction performed at different concentrations of urea. Relative initial rates (*rel. v.*) are stated. (E) Fluorescence intensity measurement of transpeptidase reaction performed at different concentrations of GuHCl. Relative initial rates are stated.

To investigate the impact of INCYPRO cross-linking, protein stability was assessed by thermal denaturation experiments (temperature range: *T* = 35–95 °C) using nano differential scanning fluorimetry (nanoDSF) as a readout. For comparison, wt SrtA and the **8M** variant were included (mass spectra Supplementary Figures S14 and S15). Cross-linked **xS11** demonstrated the highest melting temperature (*T*_i_ = 64.4 °C, Figure 3A), while its linear precursor **S11** showed the lowest stability (*T*_i_ = 48.6 °C), which was similar to enhanced SrtA variant **8M** (*T*_i_ = 52.4 °C). Interestingly, these two linear variants were considerably less stable than wt SrtA which exhibited a thermal stability slightly lower than **xS11** (*T*_i_ = 62.8 °C). This suggests that INCYPRO cross-linking can restore (and moderately surpass) the loss of stability resulting from the eight mutations required for enhanced activity. As expected, all proteins show a slightly lower stability in absence of Ca^2+^ (avg. Δ*T*_i_ = −3 °C, Supplementary Figure S16), demonstrating consistent stabilization by ion binding across the different variants.

We next assessed enzymatic activity to evaluate whether the observed increase in thermostability translates into increased activity under stressed conditions. The transpeptidation activity of the different SrtA variants was tested using a fluorophore(FITC)/quencher(Dabcyl) labeled SrtA substrate peptide (*c* = 20 μM). Assays were performed in the presence of triglycine resulting in an unquenched fluorophore (Figure 3B). Initially, activity measurements under unstressed conditions were performed confirming the expected high activity of **8M**. Importantly, cross-linked **xS11** also showed high activity, comparable to **8M** although somewhat reduced which is a trend we had already observed for the INCYPRO stabilized wt SrtA.^19^ In the absence of triglycine, SrtA triggers substrate hydrolysis. However, as expected for both **8M** and **xS11,** this activity is considerably lower than transpeptidation with triglycine (Supplementary Figure S17). Second, HPLC/MS analysis was used to assess enzymatic activity at elevated temperature (*T* = 60 °C, Figure 3C) and to confirm formation of the desired transpeptidation products (b and c, Figure 3B). For wt SrtA, **8M**, and **S11** only the presence of the starting material (a) was detected after 30 min, as one would expect under these conditions. Notably, only INCYPRO cross-linked **xS11** resulted in the formation of transpeptidation products (b and c). The identity of the corresponding HPLC signals was confirmed by MS (Supplementary Figure S18).

During purification *strategy II* (Figure 2B), we already noticed an increased folding propensity for **xS11** in the presence of the denaturant urea when compared to its linear precursor **S11**. To assess the effect on enzymatic activity, the transpeptidation assay was performed for **8M** and **xS11** in the presence of urea (*c* = 2 and 3 M, Figure 3D). Initial rates (*rel. v*, relative to **8M**) show ca. 2-fold lower activity for **xS11** under unstressed conditions. In the presence of urea, however, **xS11** performs significantly better. At 3 M urea, **8M** exhibits negligible activity (*rel. v* < 0.01), while **xS11** shows 41% of its initial activity (*rel. v* = 0.22 vs 0.54, Figure 3D). To further investigate the effect of denaturants, activity assays were performed in the presence of the strong chaotrope guanidine hydrochloride (GuHCl, *c* = 0.5 and 1 M, Figure 3E). Here, we observed an analogous trend with **8M** activity highly sensitive to GuHCl concentration. At 1 M GuHCl, **8M** does not show meaningful activity (*rel. v* < 0.01), while **xS11** exhibits 20% of its initial activity (*rel. v* = 0.11 vs 0.54, Figure 3E). Taken together, these findings confirm that the increased stability of bicyclic **xS11** also translates into relevant activity under denaturing conditions.

### Flexibility Differences in 8M and xS11

To assess if cross-linking affects the overall structure of **xS11** and to identify a possible reason for its lower activity compared to **8M** under unstressed conditions, molecular dynamics (MD) simulations were performed. Atomic models of **8M** and **xS11** were generated using an NMR structure of Ca^2+^-bound wt SrtA^34^ as a template (amino acids, aa 61–205, PDB ID: 2KID). Homology models were obtained using SWISS-MODEL,^37^ conserving the conformation of the Ca^2+^ binding site which is essential for transpeptidase activity. Both structures were equilibrated in the Amber ff14SB force field^38,39^ prior to analysis (*t* = 400 ns, Supplementary Figure S19). Notably, this equilibration did not result in significant perturbation of the tertiary structure of **8M** and **xS11**, or the position of the Ca^2+^ ion (Supplementary Figure S20). This holds particularly true for the central β-barrel, suggesting that neither the activating variations (SrtA to **8M**) nor the cysteine introduction and cross-linking (**8M** to **xS11**) have substantial effects on the overall enzyme structure.

**Figure 4:**
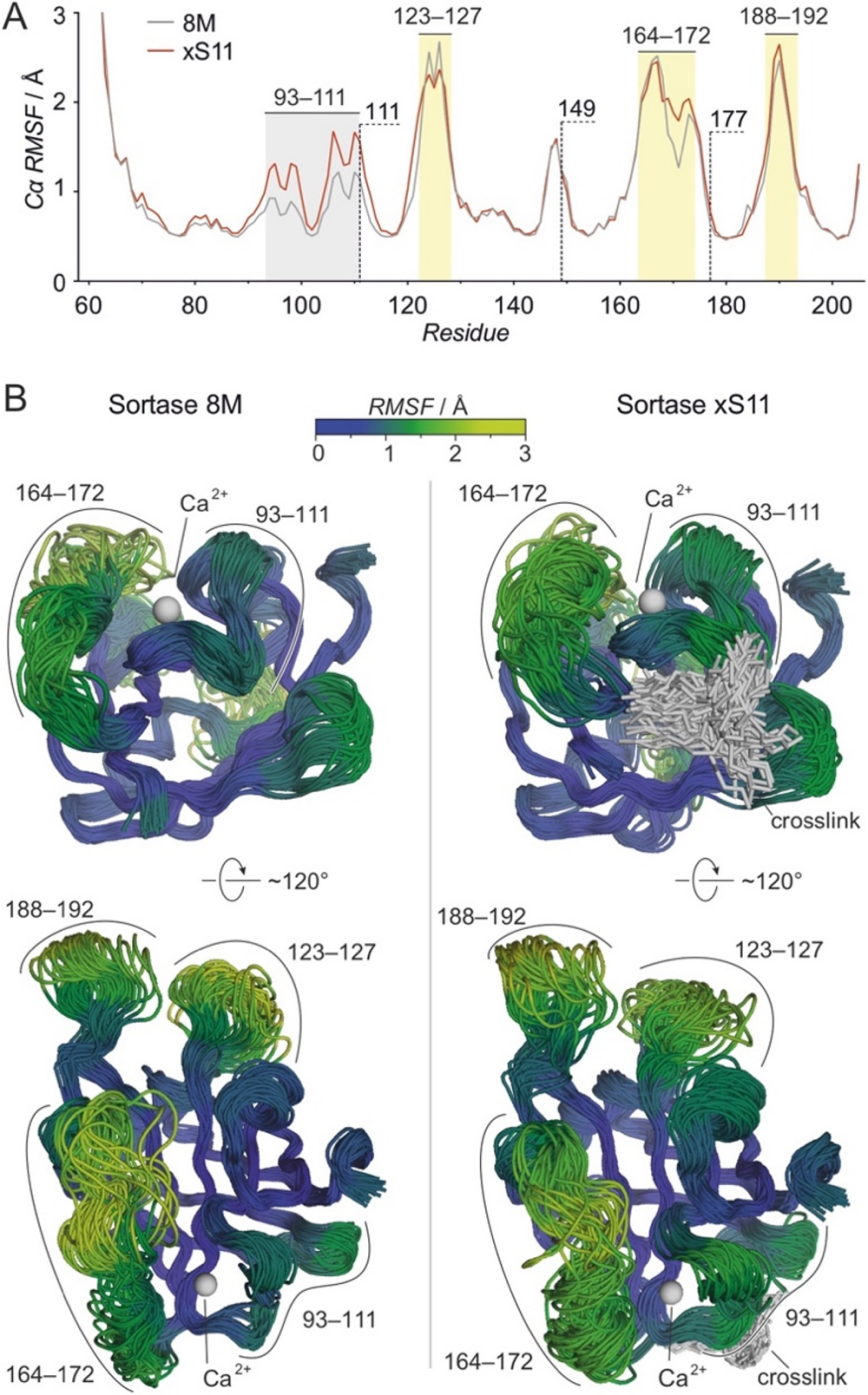
MD simulations: (A) Root mean square fluctuation (RMSF) of backbone Cα atoms (gray: **8M**, red: **xS11**) calculated from a total of 2.5 μs simulation time. Most flexible loops are highlighted (yellow) including cross-linking positions in **xS11** (111, 149, and 177). The region between residues 93–111 shows the most significant increase in flexibility upon cross-linking (gray). (B) Aligned snapshots at 50 ns intervals taken from the five, 500 ns replicates demonstrate the conformational flexibility (left: **8M**, right: **xS11**).

Subsequently, extensive MD simulations of **8M** and **xS11** were performed and analyzed across five replicate trajectories of 500 ns each (Supplementary Figure S21) aiming to analyze the effect of cross-linking on protein dynamics. For that purpose, root-mean-square fluctuation (RMSF) values were determined for α-carbon atoms of each residue (Figure 4A). Both proteins demonstrate broad flexibility in the loop regions surrounding the substrate binding site, involving residues 123–127, 164–172, and 188–192 (Figure 4A). Notably, these loops are located opposite to the cross-linking site (Figure 4B). Overall, only minor changes in backbone flexibility were observed between **8M** and **xS11**. Interestingly, slightly increased flexibility is observed in cross-linked **xS11** between residues 93–111, in a region adjacent to one of the cross-linking sites (aa 111, Figure 4A). Notably, this region is involved in Ca^2+^ binding and increased flexibility may indicate a slight perturbation of the original conformation. Since the Ca^2+^ binding site is crucial for enzymatic function, the increased flexibility may be linked to the lower activity of **xS11** under native conditions, whereas the stabilizing effects of the cross-linker are primarily observed under thermal or chemical stress.

## Conclusions

The transpeptidase SrtA represents a useful tool for protein modification. However, wt SrtA exhibits relatively low activity which hampers its applicability. Activity enhancing variants have been generated, such as octa mutant **8M**,^30^ however, at the cost of considerably reduced thermal stability (Δ*T*_i_ = −10 °C). This limits the application range of such activity-enhanced SrtA variants. Based on the **8M** variant, we generated a stable and active SrtA version (**xS11**) using the INCYPRO technology. This involves the introduction of three spatially aligned cysteine residues which are cross-linked using a triselectrophilic reagent (Ae2). The resulting bicyclic **xS11** indeed shows high activity and a thermal stability (*T*_i_ = 64.4 °C) exceeding **8M** (*T*_i_ = 52.4 °C) and wt SrtA (*T*_i_ = 62.8 °C). Modeling of **8M** and **xS11** verify for both an overall fold very similar to wt SrtA. Interestingly, MD simulations suggest for one region in **xS11** a higher flexibility than in **8M** which may be due to structural perturbations by cysteine introduction. This is supported by the lower thermal stability of linear precursor and triple cysteine variant **S11** when compared to **8M** (Δ*T*_i_ = −3 °C) and may then also explain the slightly reduced activity of **S11** and **xS11** compared to **8M** under unstressed conditions. Notably, under thermal or chemical stress, **xS11** outperforms **8M** due to the overall increased stability of its tertiary structure. This unique feature now opens the door to entirely new applications allowing the enzymatic modification of protein substrates under denaturing conditions.

## Materials and Methods

### Protein Expression

Chemically competent *E. coli* cells were transformed with a *pET28* vector containing the genes coding for His_6_-tagged Sortase A and variants **8M** and **S11**. Cells were grown in TB medium at 37 °C, and protein expression was induced with 0.5 mM IPTG and performed at 25 °C overnight. Cells were harvested using centrifugation (Beckman Coulter JLA8.1, 4000 rpm, 20 min, 4 °C) and resuspended in lysis buffer (20 mM HEPES, pH 7.5, 150 mM NaCl, 5 mM CaCl_2_, 0.5 mM TCEP). Cell lysis was performed using a microfluidizer (Microfluidics LM10) at 15000 psi. The resulting cell lysate was cleared of debris by centrifugation (Beckman Coulter JA25.50, 21000 rpm, 45 min, 4 °C). Cleared cell lysate was loaded on a pre-equilibrated Ni-affinity column (Cytiva, HisTrap FF crude 5 mL). The column was washed with eight column volumes (CV) of wash buffer (50 mM HEPES, pH 7.5, 400 mM NaCl, 5% glycerol, 20 mM imidazole, 0.5 mM DTT) and protein eluted by circulating PreScission protease overnight for on-column digest. Eluted protein was subjected to size exclusion chromatography (SEC) (GE Healthcare, Äkta Pure, HiLoad 16/600 S75) using SEC buffer (20 mM HEPES, pH 7.5, 150 mM NaCl, 0.5 mM TCEP). Protein containing fractions were pooled, concentrated (3 kDa MWCO amicon centrifugal filter, Merck), and flash frozen in liquid nitrogen. Sortase A wild type was purified as previously reported.^19^

### INCYPRO Cross-Linking and MS Characterization

Tris-electrophile Ae2 was synthesized as previously reported.^28^ Cross-linking of **S11** with Ae2 was performed in cross-linking buffer (50 mM HEPES, pH 8.5, 50 mM NaCl) in the absence or presence of 5 mM CaCl_2_. A 100 mM stock solution of Ae2 in DMSO was diluted to 2 mM with cross-linking buffer. For the reaction, 100 μM protein and 1 mM Ae2 were incubated in a total volume of 200 μL cross-linking buffer for 3.5 h at 25 °C. For purification procedures, see below. Protein mass spectra were obtained on an Agilent HPLC/ESI-MS system (1260/1290 Infinity, 6120 Quadrupole) with a Nucleodur C4 5 μm, 125 mm × 4.0 mm column (solvents: H_2_O with 0.1% formic acid and 0.01% TFA; acetonitrile with 0.1% formic acid and 0.01% TFA).

### Synthesis of Biotinylated Thiol

Biotinylated thiol biotin-PEG_3_-Cys was synthesized using Fmoc-based solid-phase peptide synthesis.^40,41^ In brief, the H-Rink amide resin was used for the synthesis and all couplings were performed in an orthogonal manner. Removal of the Fmoc-group was performed with 25% piperidine in DMF for 10 min. First coupling was performed using 4 equiv of building block, 4 equiv of COMU, 4 equiv of OxymaPure, and 8 equiv of DIPEA in DMF for 30 min. Second coupling was done with 4 equiv building block, 4 equiv PyBOP, and 8 equiv DIPEA in DMF for 30 min. Residual free amines were acetylated using Ac_2_O/DIPEA/DMF (1/1/8) for 5 min. Final cleavage from resin and removal of protecting groups was performed using four times 1 mL TFA/TIPS/ODT/H_2_O (94/1/2.5/2.5). TFA was evaporated and the thiol was precipitated by the addition of H_2_O. Subsequently, the sample was flash-frozen and lyophilized. Final purification was performed using a Nucleodur C18 Gravity 5 μm column on an Agilent HPLC system with solvents H_2_O (with 0.1% TFA) and acetonitrile (with 0.1% TFA). Characterization of the product was performed on an Agilent HPLC/MS system with a Nucleodur C4 5 μm column.

### Purification Strategy I

Cross-linking of **S11** with Ae2 was performed as described above. Then, Ae2 was removed from the reaction solution by using a centrifugal concentrator (VivaSpin500, 3 kDa MWCO, Sartorius) and washing with cross-link buffer. 1.75 mM biotin-PEG_3_-Cys were added to the protein solution in a total of 400 μL cross-linking buffer and the reaction mixture was incubated at 25 °C for 3.5 h. Following that, the remaining excess of biotin-PEG_3_-Cys was removed using a centrifugal concentrator (VivaSpin500, 3 kDa MWCO, Sartorius) and buffer2 (50 mM HEPES, pH 7.5, 50 mM NaCl). Thereafter, the reaction mixture was incubated with streptavidin beads (200 μL, 800 pmol/mL capacity, Thermo Fisher) and incubated at 4 °C for 1 h. The supernatant containing **xS11** was collected, concentrated using a centrifugal concentrator (VivaSpin500, 3 kDa MWCO, Sartorius), analyzed via MS (as above), flash-frozen in liquid nitrogen, and stored at −80 °C.

### Purification Strategy II

Cross-linking of **S11** with Ae2 was performed in cross-link buffer (50 mM HEPES, pH 8.5, 50 mM NaCl). In a total of 200 μL, 200 μM **S11** and 1 mM Ae2 were diluted and incubated for 2 h at 25 °C. Thereafter, the reaction mixture was reduced using a centrifugal concentrator (VivaSpin500, 5 kDa MWCO, Sartorius) to 90 μL and 400 μL of SEC buffer (20 mM HEPES, pH 7.5, 150 mM NaCl, 5 mM CaCl_2_, 2.5 M urea) were added. The sample was injected on a Superdex75 10/300 GL column (Äkta Pure, GE Healthcare) running on the same buffer. Target protein containing fractions were collected and buffer exchanged on the same column using SEC buffer (20 mM HEPES, pH 7.5, 150 mM NaCl, 5 mM CaCl_2_). Then, protein containing fractions were pooled, concentrated using a centrifugal concentrator (VivaSpin500, 3 kDa MWCO, Sartorius), analyzed via MS, flash-frozen in liquid nitrogen, and stored at −80 °C.

### Thermal Denaturation Experiments

Thermal stability of sortase variants was assessed by nano differential scanning fluorimetry (nanoDSF), measuring the intrinsic fluorescence ratio at 350 nm/330 nm. Data was collected on a Tycho NT.6 instrument (NanoTemper) from 35–95 °C, with a ramp rate of 30 °C min^–1^ and a measurement interval of 1 °C. Samples were measured at a protein concentration of 5–10 μM buffered at pH 7.5 in 20 mM HEPES, 150 mM NaCl, with 0 or 5 mM CaCl_2_. The first derivative of the ratio was plotted as a function of temperature, and the inflection temperature (*T*_*i*_) determined as the peak of the derivative curve.

### Transpeptidase Activity Assay

The assay was performed in assay buffer (20 mM HEPES, pH 7.5, 150 mM NaCl, 5 mM CaCl_2_, 0.01% Tween20, 0.5 mM TCEP). SrtA variants were prediluted to 50 μM with assay buffer lacking Tween20 and TCEP, and finally diluted to 6 μM in assay buffer. The fluorescently labeled probe was obtained as reported previously^19^ and dissolved to 100 μM in assay buffer. Triglycine (H-GGG-OH) was dissolved to 10 mM in assay buffer. In a Corning 384 black U-bottom plate, 3 μL probe solution, 3 μL triglycine solution, and 4 μL assay buffer were mixed and the reaction was started by the addition of 5 μL enzyme solution (final concentrations: 2 μM enzyme, 20 μM probe, and 2 mM triglycine). Additionally, 3 μL probe solution and 3 μL triglycine solution were mixed with 9 μL assay buffer to serve as a blank. For normalization, 3 μL of a 200 μM solution of GEK-FITC was mixed with 12 μL assay buffer, and the resulting fluorescence intensity defined as 100%. Fluorescence intensities were measured with a Tecan Spark 20M plate reader (λ_Ex_= 475 nm, λ_Em_= 520 nm) every 30 s. For transpeptidase activity assays in the presence of denaturants, the given amount of urea or GuHCl was added to the assay buffer and samples were prepared as mentioned above. HPLC/MS readout was performed on an Agilent HPLC/MS system with a Nucleodur C4 5 μm column, with solvent A: H_2_O (with 0.1% TFA) and solvent B: acetonitrile (with 0.1% TFA, gradient 20–95% B).

### Molecular Dynamics (MD) Simulations

The calcium-bound NMR structure of wt SrtA (PDB ID: 2KID) was used as a template to generate homology models of **8M** and **xS11** with SWISS-MODEL,^37^ and the initial structure of the covalently attached cross-linker was built and minimized using Avogadro.^42^ MD simulations were performed using Amber20.^39,43^ The cross-linker was parametrized using the general Amber force field (GAFF); partial charges were calculated using antechamber^39^ by the AM1-BCC method^44^ with thiol groups capping all carbon atoms involved in bonding to cysteine side chains. Partial charges were refitted across the uncapped Ae2 molecule using *prepgen* to generate the final parameters (ae2.frcmod, ae2.lib), and cross-linked cysteines defined as CYX residues. The ff14SB force field^38^ was applied for protein parametrization. Ca^2+^-bound structures of **8M** and **xS11** were prepared using *tleap* by solvation in a TIP3P water box (with a 12 Å minimum protein-edge distance) and neutralized by addition of chloride ions. Each system was minimized and heated to 300 K, followed by a 200 ps NPT equilibration and 500 ns production MD using the *pmemd.cuda* application. Simulations were performed using the VU Bazis Computational Cluster. Trajectories were analyzed using *cpptraj*, with Cα root-mean-square fluctuation (RMSF) values calculated relative to the equilibrated snapshot at 400 ns from the initial trajectory (Supplementary Figure 19). All parameters, input files, and protein structures are available in the following GitHub repository: https://github.com/georgehutch/xS11_INCYPRO.

## Supporting Information Available

Supplementary Figures (PDF)
PDB file with atomic coordinates of **8M** model (PDB)
PDB file with atomic coordinates of **S11** model (PDB)
PDB file with atomic coordinates of **xS11** model (PDB)

## Supplementary Material

bc3c00151_si_001.pdf

bc3c00151_si_002.pdb

bc3c00151_si_003.pdb

bc3c00151_si_004.pdb
