# Supplementary material for: Bicyclic Engineered Sortase A Performs Transpeptidation under Denaturing Conditions
Source: Bioconjug Chem. 2023 May 29;34(6):1114–21. doi: 10.1021/acs.bioconjchem.3c00151 (PMC10288436; doi:10.1021/acs.bioconjchem.3c00151)
Supplement: Supplementary file 1 — bc3c00151_si_001.pdf [file bc3c00151_si_001.pdf]

Supplementary Information

Bicyclic engineered Sortase A performs transpeptidation under denaturing conditions

Sebastian Kiehstaller,[1] George H. Hutchins,[2,3] Alessia Amore,[2,3] Alan Gerber,[2,3] Mohamed Ibrahim,[2] Sven Hennig, [2,3] Saskia Neubacher, [1,*] Tom N. Grossmann[2,3,*]

[1] Incircular BV, De Boelelaan 1108, 1081 HZ Amsterdam, The Netherlands.

[2] Department of Chemistry & Pharmaceutical Sciences, Vrije Universiteit Amsterdam, De Boelelaan 1083, 1081 HV, Amsterdam, The Netherlands.

[3] Amsterdam Institute of Molecular and Life Sciences, Vrije Universiteit Amsterdam, De Boelelaan 1083, 1081 HV, Amsterdam, The Netherlands.

* corresponding authors, emails: saskia@incircular.com, t.n.grossmann@vu.nl

Supplementary Figures

```
        60        70        80        90        100       110
SrtA    QAKPQIPKDKSKVAGYIEIPDADIKEPVYPGPATPEQLNRGVSFAEENESLDDQNISIAG
8M      QAKPQIPKDKSKVAGYIEIPDADIKEPVYPGPATREQLNRGVSFAEENESLDDQNISIAG
xS11    QAKPQIPKDKSKVAGYIEIPDADIKEPVYPGPATREQLNRGVSFAEENESLCDQNISIAG

        120       130       140       150       160       170
        HTFIDRPNYQFTNLKAAKKGSMVYFKVGNETRKYKMTSIRDVKPTDVGVLDEQKGKDKQL
        HTFIGRPNYQFTNLKAAKKGSMVYFKVGNETRKYKMTSIRNVKPTAVGVLDEQKGKDKQL
        HTFIGRPNYQFTNLKAAKKGSMVYFKVGNCTRKYKMTSIRNVKPTAVGVLDEQKGKDCQL

        180       190       200
        TLITCDDYNEKTGVWEKRKIFVATEVK
        TLITCDDLNRETGVWETRKIFVATEVK
        TLITCDDLNRETGVWETRKIFVATEVK
```

Figure S1: Amino acid sequence alignment of **SrtA**, **8M** and **S11** from residue 60 – 206. The eight activity enhancing residues are indicated in blue, and the three additional introduced cysteines for crosslinking in red.

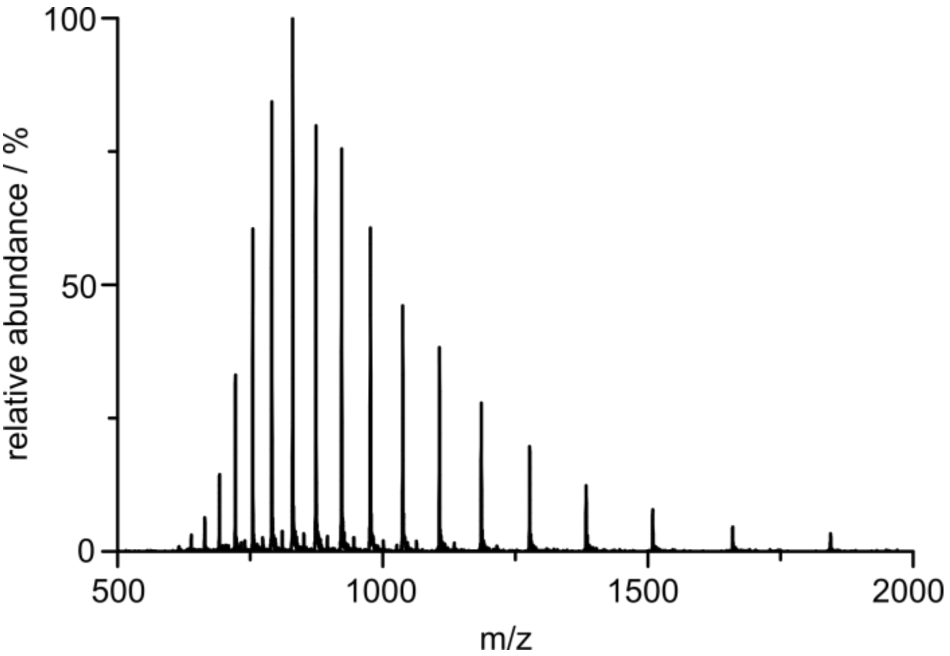

MW (**S11**): calculated = 16594.98 Da
found = 16593.09 Da (deconvolution)

	m/z calculated	m/z found
$[M+11H]^{+11}$	1509.63	1509.4
$[M+12H]^{+12}$	1383.92	1384.0
$[M+13H]^{+13}$	1277.54	1277.4
$[M+14H]^{+14}$	1186.36	1186.3
$[M+15H]^{+15}$	1107.33	1107.3
$[M+16H]^{+16}$	1038.19	1038.1
$[M+17H]^{+17}$	977.18	977.1
$[M+18H]^{+18}$	922.94	922.9
$[M+19H]^{+19}$	874.42	874.4
$[M+20H]^{+20}$	830.75	830.7
$[M+21H]^{+21}$	791.24	791.2
$[M+22H]^{+22}$	755.32	755.2
$[M+23H]^{+23}$	722.52	722.5
$[M+24H]^{+24}$	692.46	692.4
$[M+25H]^{+25}$	664.80	664.9

Figure S2: MS spectrum of **S11** including calculated and found m/z signals.

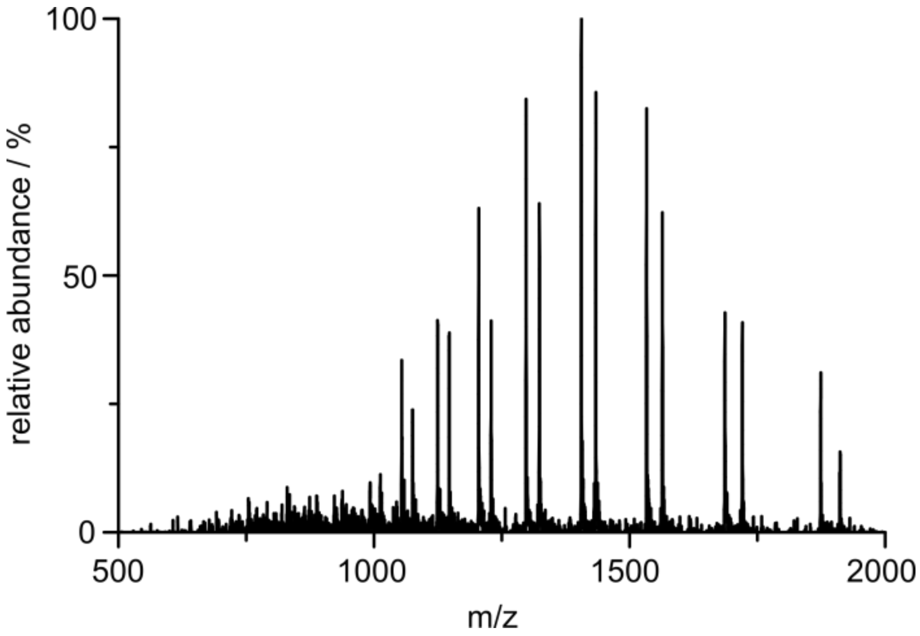

MW(**xS11**): calculated = 16861.33 Da
found = 16859.18 Da (deconvolution)

MW(**oS11**): calculated = 17200.33 Da
found = 17198.92 Da (deconvolution)

	**xS11**		**oS11**	
	m/z calculated	m/z found	m/z calculated	m/z found
$[M+9H]^{+9}$	1874.48	1874.4	1912.15	1911.5
$[M+10H]^{+10}$	1687.13	1686.8	1721.03	1720.9
$[M+11H]^{+11}$	1533.85	1533.6	1564.67	1564.5
$[M+12H]^{+12}$	1406.11	1405.9	1434.36	1434.3
$[M+13H]^{+13}$	1298.03	1297.9	1324.10	1324.0
$[M+14H]^{+14}$	1205.38	1205.2	1229.60	1229.5
$[M+15H]^{+15}$	1125.09	1124.9	1147.6	1147.4
$[M+16H]^{+16}$	1054.83	1054.5	1076.02	1075.8

Figure S3: MS spectrum of reaction of **S11** with Ae2 in the presence of $CaCl_2$ including calculated and found m/z signals.

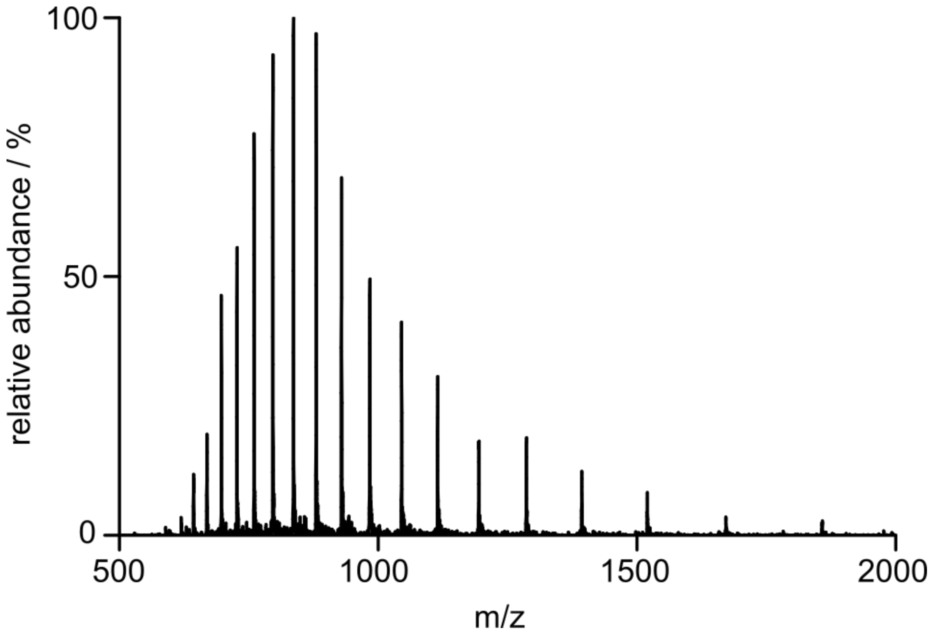

MW(**8M**-IAA): calculated = 16715.01 Da

found = 16713.50 Da (deconvolution)

	m/z calculated	m/z found
$[M+11H]^{+11}$	1520.55	1520.6
$[M+12H]^{+12}$	1393.92	1993.9
$[M+13H]^{+13}$	1286.77	1286.8
$[M+14H]^{+14}$	1194.93	1194.6
$[M+15H]^{+15}$	1115.33	1115.2
$[M+16H]^{+16}$	1045.69	1045.6
$[M+17H]^{+17}$	984.24	984.2
$[M+18H]^{+18}$	929.61	929.5
$[M+19H]^{+19}$	880.74	8807
$[M+20H]^{+20}$	836.75	836.7
$[M+21H]^{+21}$	796.95	796.9
$[M+22H]^{+22}$	760.77	760.8
$[M+23H]^{+23}$	727.74	727.6
$[M+24H]^{+24}$	697.46	697.2
$[M+25H]^{+25}$	669.60	669.7
$[M+26H]^{+26}$	643.89	643.5

Figure S4: MS spectrum of **8M** after treatment with iodoacetamide (IAA). Deconvolution shows **8M** reacting with one molecule IAA. MS-Chromatograms including calculated and found m/z signals.

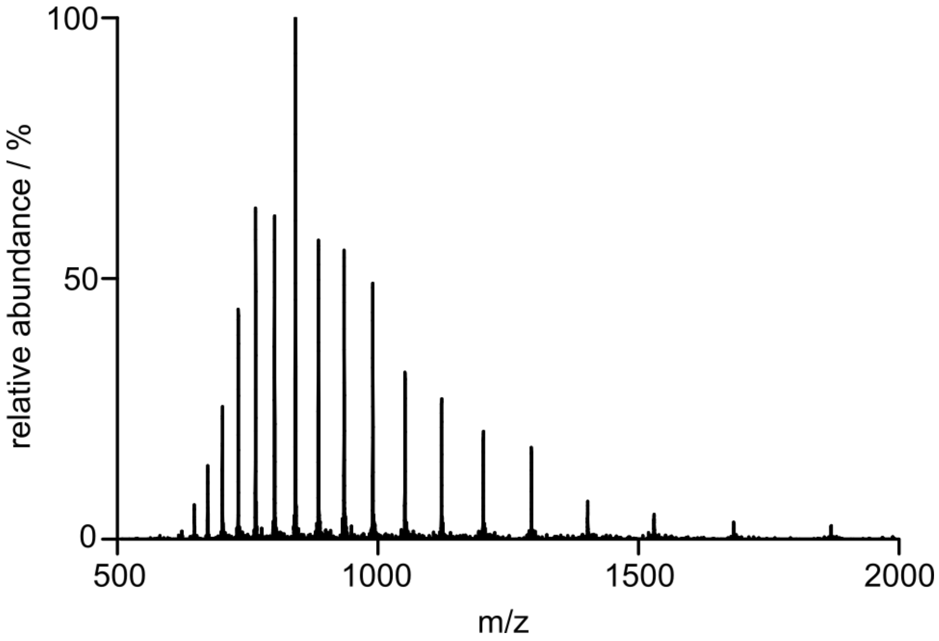

MW(**S11**-(4xIAA)): calculated = 16823.26 Da

found = 16820.92 Da (deconvolution)

	m/z calculated	m/z found
$[M+12H]^{+12}$	1402.94	1402.9
$[M+13H]^{+13}$	1295.10	1294.9
$[M+14H]^{+14}$	1202.66	1202.6
$[M+15H]^{+15}$	1122.55	1122.5
$[M+16H]^{+16}$	1052.45	1052.4
$[M+17H]^{+17}$	990.60	990.6
$[M+18H]^{+18}$	935.63	935.5
$[M+19H]^{+19}$	886.43	886.3
$[M+20H]^{+20}$	842.16	842.1
$[M+21H]^{+21}$	802.11	802.0
$[M+22H]^{+22}$	765.69	765.6
$[M+23H]^{+23}$	732.45	732.4
$[M+24H]^{+24}$	701.97	701.9
$[M+25H]^{+25}$	673.93	673.8
$[M+26H]^{+26}$	648.05	648.0

Figure S5: MS spectrum of **S11** after treatment with iodoacetamide (IAA). Deconvolution shows **S11** reacting with 4 molecules of IAA. MS-Chromatograms including calculated and found m/z signals.

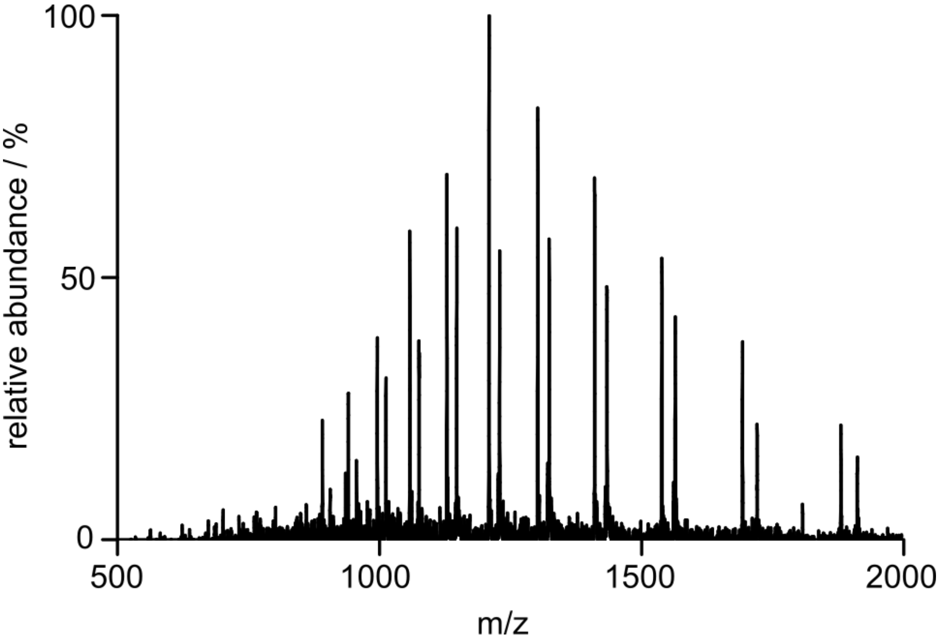

MW(**xS11**-IAA): calculated = 16918.40 Da
found = 16916.44 Da (deconvolution)

MW(**oS11**): calculated = 17200.33 Da
found = 17198.77 Da (deconvolution)

	**xS11-IAA**		**oS11**	
	m/z calculated	m/z found	m/z calculated	m/z found
$[M+9H]^{+9}$	1880.82	1880.6	1912.15	1911.9
$[M+10H]^{+10}$	1692.84	1692.7	1721.03	1720.8
$[M+11H]^{+11}$	1539.04	1639.1	1564.67	1564.7
$[M+12H]^{+12}$	1410.87	1410.6	1434.36	1434.1
$[M+13H]^{+13}$	1302.42	1302.3	1324.10	1324.0
$[M+14H]^{+14}$	1209.46	1209.3	1229.60	1229.4
$[M+15H]^{+15}$	1128.89	1128.7	1147.6	1147.7
$[M+16H]^{+16}$	1058.40	1058.2	1076.02	1076.1
$[M+17H]^{+17}$	996.20	996.1	1012.78	1012.5
$[M+18H]^{+18}$	940.91	940.9	956.57	956.4
$[M+19H]^{+19}$	891.44	891.4	906.28	906.3

Figure S6: MS spectrum of a mixture of **oS11** and **xS11** after treatment with iodoacetamide (IAA). Deconvolution shows **xS11** with one molecule IAA, while **oS11** does not react with IAA. MS-Chromatograms including calculated and found m/z signals.

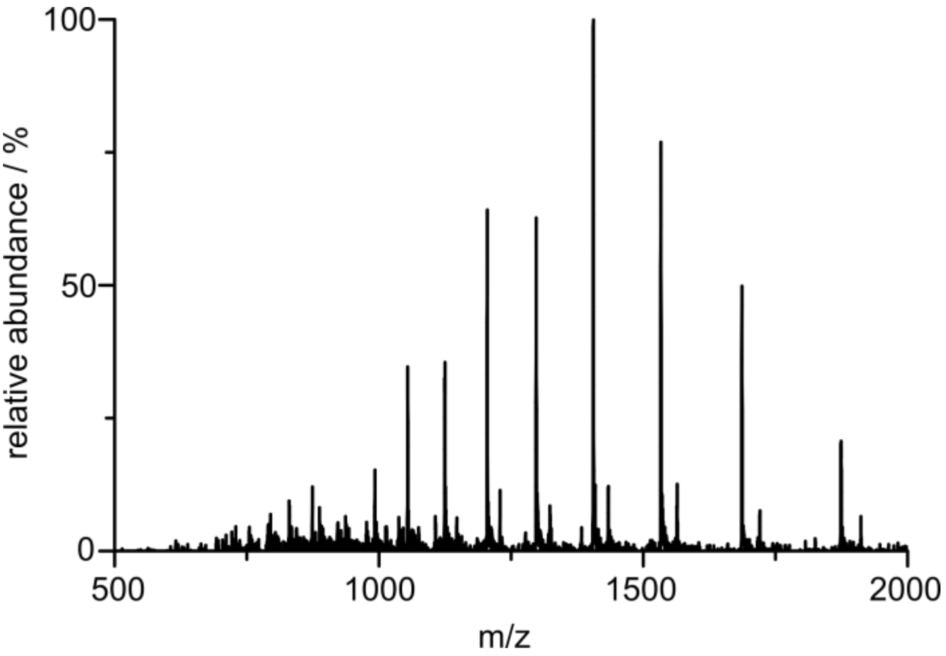

MW(**xS11**): calculated = 16861.33 Da
found = 16859.18 Da (deconvolution)

MW(**oS11**): calculated = 17200.33 Da
found = 17198.50 Da (deconvolution)

	**xS11**		**oS11**	
	m/z calculated	m/z found	m/z calculated	m/z found
$[M+10H]^{+10}$	1687.13	1686.8	1721.03	1721.0
$[M+11H]^{+11}$	1533.85	1533.7	1564.67	1564.6
$[M+12H]^{+12}$	1406.11	1405.9	1434.36	1434.4
$[M+13H]^{+13}$	1298.03	1297.9	1324.10	1323.9
$[M+14H]^{+14}$	1205.38	1205.5	1229.60	1229.4
$[M+15H]^{+15}$	1125.09	1124.8	1147.6	1147.6
$[M+16H]^{+16}$	1054.83	1054.7		
$[M+17H]^{+17}$	992.84	992.7		

Figure S7: MS spectrum of reaction of **S11** with Ae2 in the absence of $CaCl_2$ including calculated and found m/z signals.

biotin-PEG$_3$-Cys (**B3C**)

MW = 549.70 Da

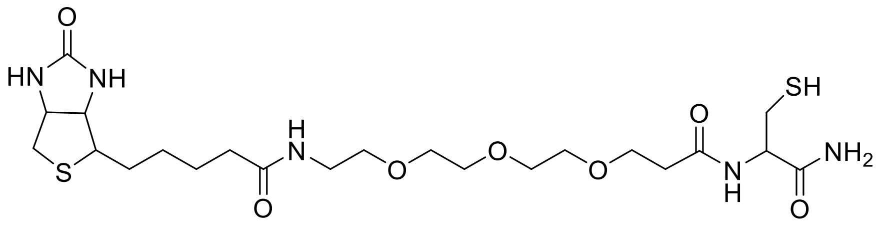

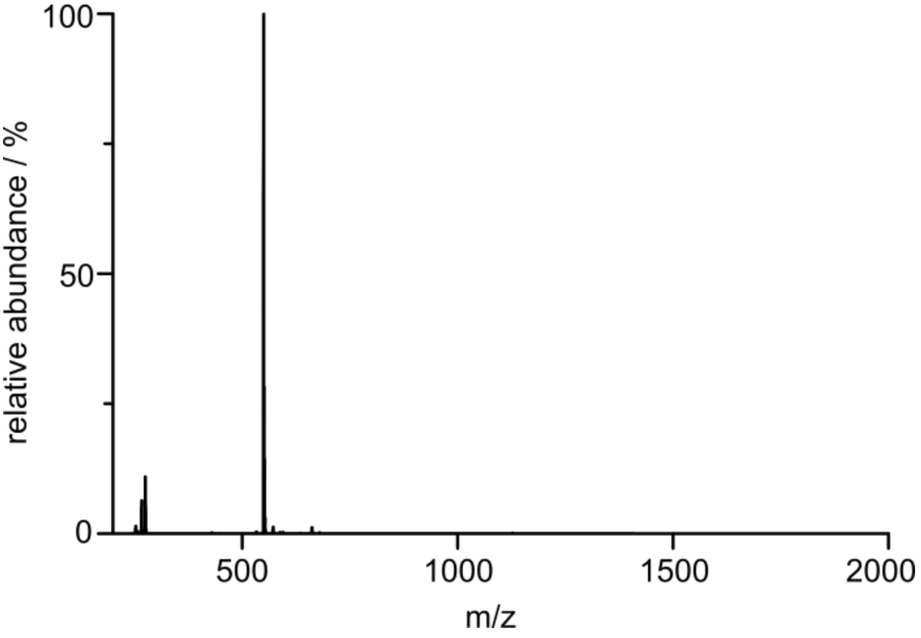

	m/z calculated	m/z found
$[M+1H]^{+1}$	550.7	550.2

Figure S8: MS spectrum of reaction of biotinylated thiol (**B3C**) including calculated and found m/z signals.

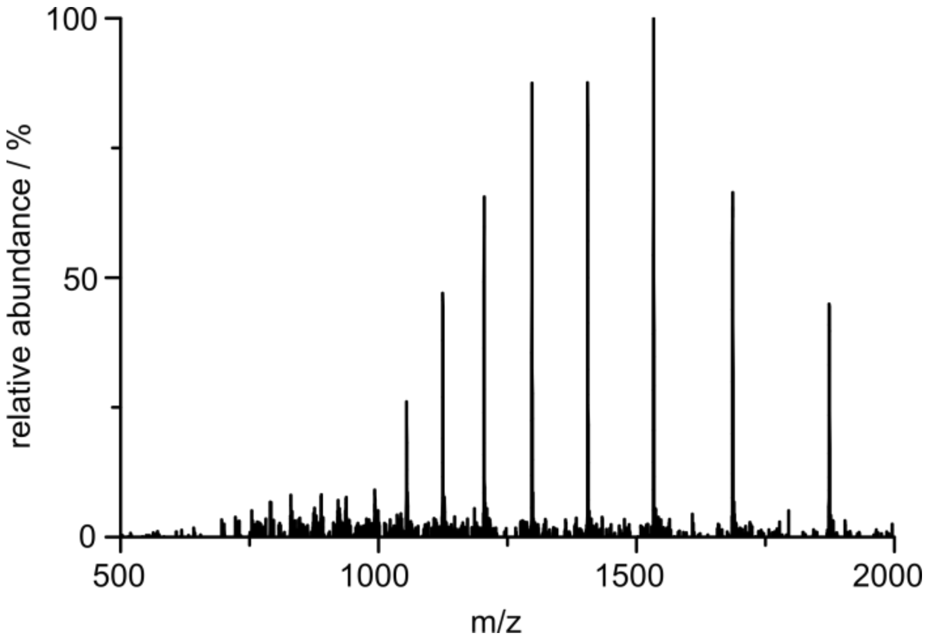

MW(**xS11**): calculated = 16861.33 Da
found = 16858.56 Da (deconvolution)

	m/z calculated	m/z found
$[M+9H]^{+9}$	1874.48	1874.1
$[M+10H]^{+10}$	1687.13	1686.7
$[M+11H]^{+11}$	1533.85	1533.7
$[M+12H]^{+12}$	1406.11	1405.7
$[M+13H]^{+13}$	1298.03	1297.8
$[M+14H]^{+14}$	1205.38	1205.2
$[M+15H]^{+15}$	1125.09	1124.9
$[M+16H]^{+16}$	1054.83	1054.8

Figure S9: MS spectrum of **xS11** purified by scavenger-based pulldown (Strategy I) including calculated and found m/z signals.

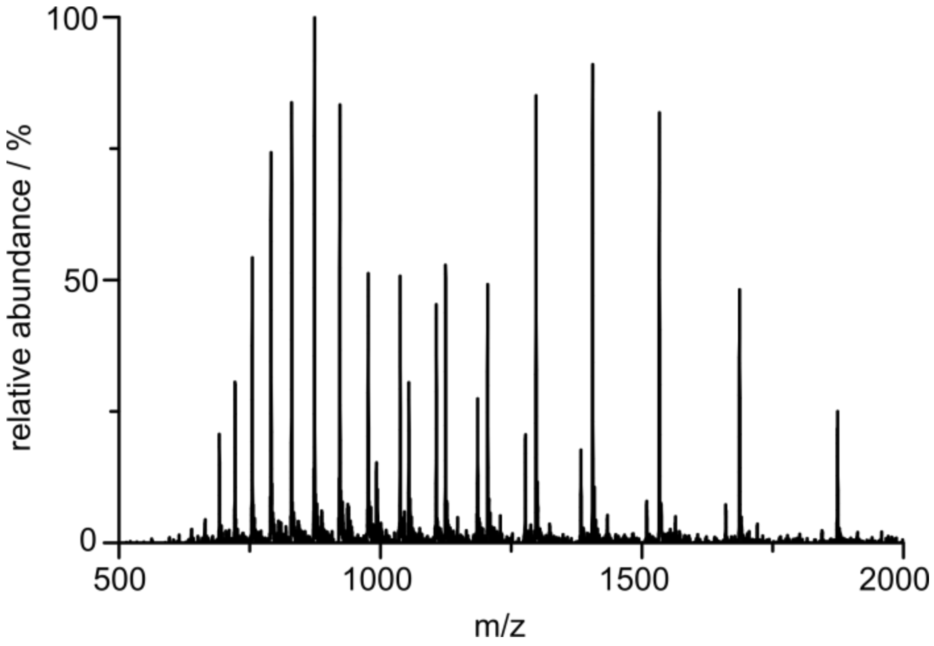

MW(**S11**): calculated = 16594.98 Da
found = 16592.42 Da (deconvolution)

MW(**xS11**) calculated = 16861.33 Da
found = 16859.55 Da (deconvolution)

	**S11**		**xS11**	
	m/z calculated	m/z found	m/z calculated	m/z found
$[M+9H]^{+9}$			1874.48	1874.5
$[M+10H]^{+10}$			1687.13	1687.1
$[M+11H]^{+11}$			1533.85	1533.6
$[M+12H]^{+12}$			1406.11	1406.0
$[M+13H]^{+13}$	1277.54	1277.5	1298.03	1297.9
$[M+14H]^{+14}$	1186.36	1186.2	1205.38	1205.2
$[M+15H]^{+15}$	1107.33	1107.2	1125.09	1124.9
$[M+16H]^{+16}$	1038.19	1038.1	1054.83	1054.6
$[M+17H]^{+17}$	977.18	977.1	922.84	992.6
$[M+18H]^{+18}$	922.94	922.8		
$[M+19H]^{+19}$	874.42	874.3		
$[M+20H]^{+20}$	830.75	830.7		
$[M+21H]^{+21}$	791.24	794.1		
$[M+22H]^{+22}$	755.32	755.2		
$[M+23H]^{+23}$	722.52	722.4		
$[M+24H]^{+24}$	692.46	692.3		

Figure S10: MS spectrum of incomplete reaction of **S11** with Ae2 including calculated and found m/z signals.

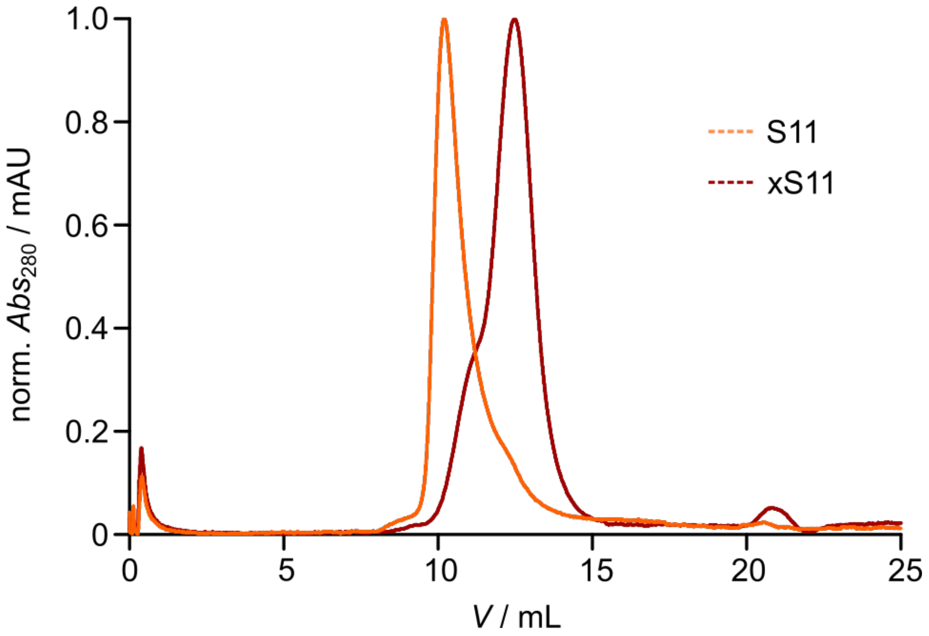

Figure S11: Overlay of normalized SEC chromatograms of **S11** and **xS11** in denaturating SEC using 2.5 M urea. A shift in retention by unfolding between **S11** and **xS11** enables the separation of the two molecules.

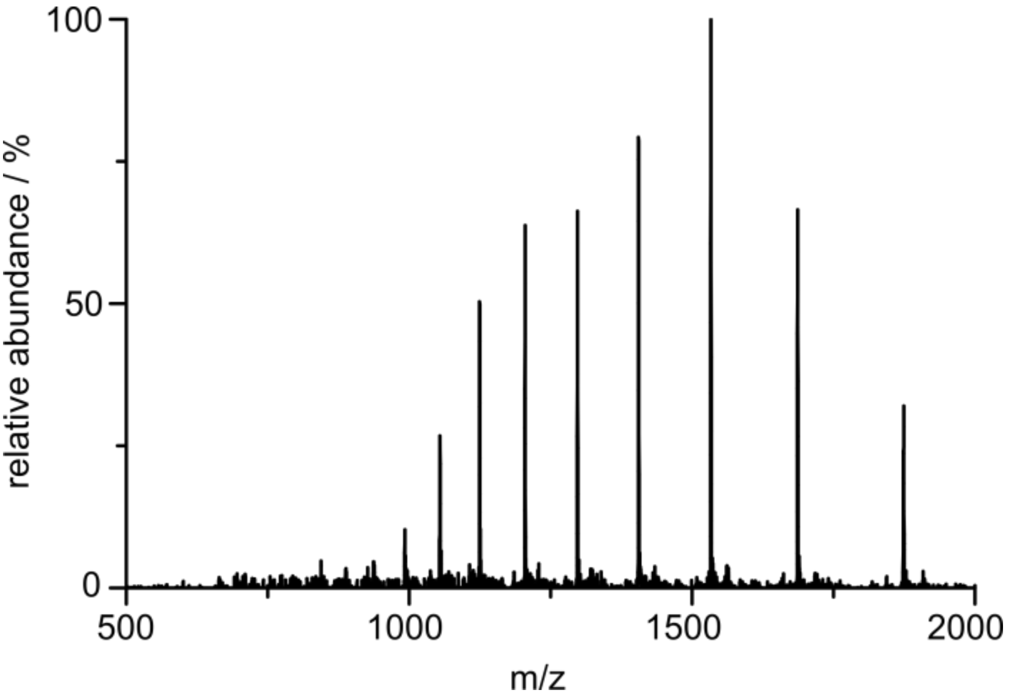

MW(**xS11**): calculated = 16861.33 Da

found = 16859.50 Da (deconvolution)

	m/z calculated	m/z found
$[M+9H]^{+9}$	1874.48	1874.4
$[M+10H]^{+10}$	1687.13	1687.0
$[M+11H]^{+11}$	1533.85	1533.7
$[M+12H]^{+12}$	1406.11	1405.8
$[M+13H]^{+13}$	1298.03	1298.0
$[M+14H]^{+14}$	1205.38	1205.2
$[M+15H]^{+15}$	1125.09	1124.9
$[M+16H]^{+16}$	1054.83	1054.8
$[M+17H]^{+17}$	992.48	993.6

Figure S10: MS spectrum of **xS11** purified by denaturating SEC (Strategy B) including calculated and found m/z signals.

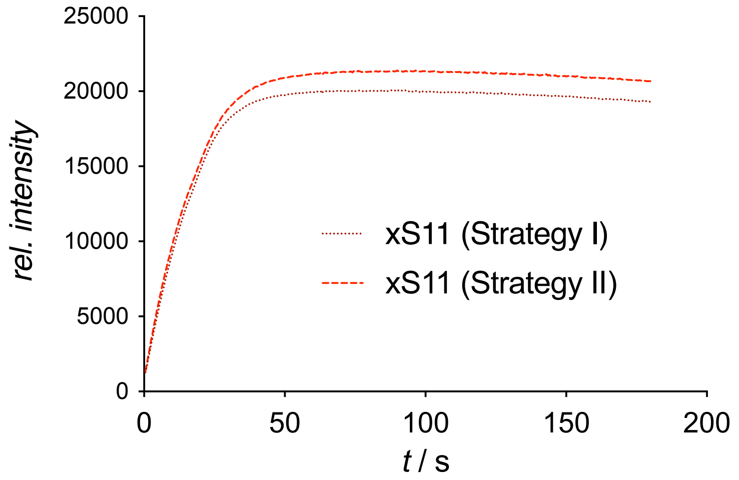

Figure S13: Fluorescence-based readout of transpeptidase activity of **xS11** purified by both strategies (biotin pulldown and denaturing size exclusion) under unstressed conditions. 2 µM protein was mixed with 20 µM probe and 2 mM triglycine (H-GGG-OH). Increase in FITC fluorescence was measured over time in a plate reader.

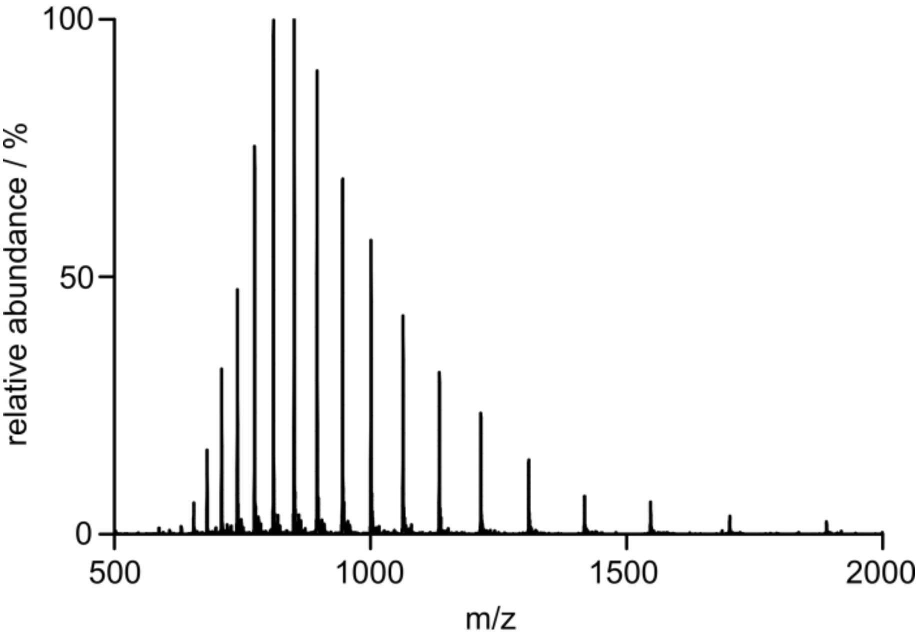

MW(SrtA): calculated = 17009.27 Da

found = 17007.39 Da (deconvolution)

	MS-ions calculated	MS-ions found
$[M+11H]^{+11}$	1547.30	1547.2
$[M+12H]^{+12}$	1418.44	1418.4
$[M+13H]^{+13}$	1309.41	1309.2
$[M+14H]^{+14}$	1215.95	1215.8
$[M+15H]^{+15}$	1134.95	1134.9
$[M+16H]^{+16}$	1064.08	1064.0
$[M+17H]^{+17}$	1001.55	1001.4
$[M+18H]^{+18}$	945.96	945.9
$[M+19H]^{+19}$	896.22	896.2
$[M+20H]^{+20}$	851.46	851.4
$[M+21H]^{+21}$	810.97	810.9
$[M+22H]^{+22}$	774.15	774.1
$[M+23H]^{+23}$	740.53	740.4
$[M+24H]^{+24}$	709.72	709.7
$[M+25H]^{+25}$	681.37	681.3

Figure S14: MS spectrum of **SrtA** including calculated and found m/z signals.

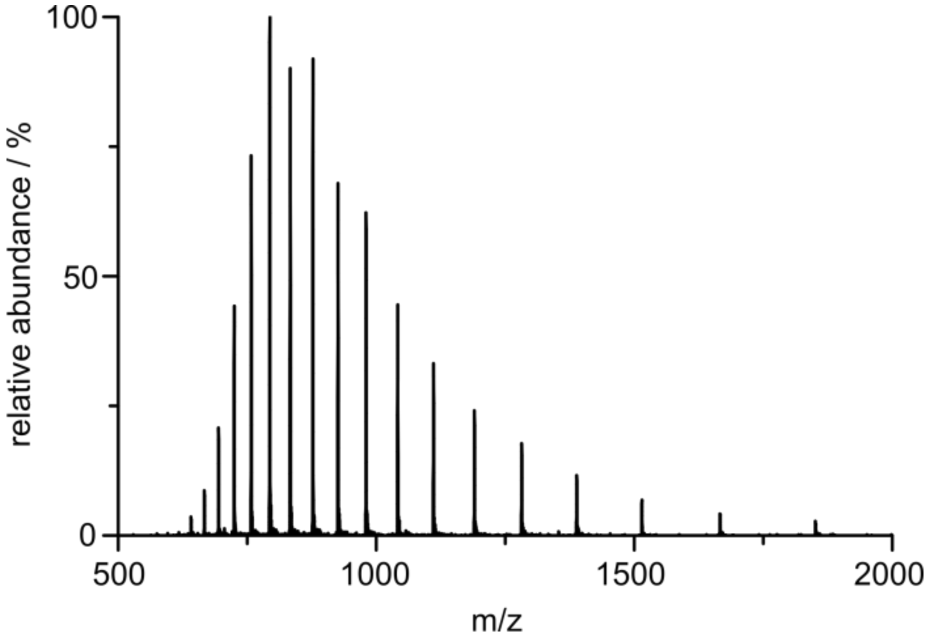

MW(**8M**): calculated = 16657.94 Da

found = 16655.92 Da (deconvoluation)

	m/z calculated	m/z found
$[M+11H]^{+11}$	1515.36	1515.2
$[M+12H]^{+12}$	1389.16	1389.0
$[M+13H]^{+13}$	1282.38	1282.4
$[M+14H]^{+14}$	1190.85	1190.7
$[M+15H]^{+15}$	1111.53	1111.4
$[M+16H]^{+16}$	1042.12	1042.1
$[M+17H]^{+17}$	980.88	980.8
$[M+18H]^{+18}$	926.44	926.3
$[M+19H]^{+19}$	877.73	877.6
$[M+20H]^{+20}$	833.90	833.9
$[M+21H]^{+21}$	794.24	794.2
$[M+22H]^{+22}$	758.18	758.1
$[M+23H]^{+23}$	725.26	725.2
$[M+24H]^{+24}$	695.08	694.8
$[M+25H]^{+25}$	667.32	667.3

Figure S15: MS spectrum of **8M** including calculated and found m/z signals.

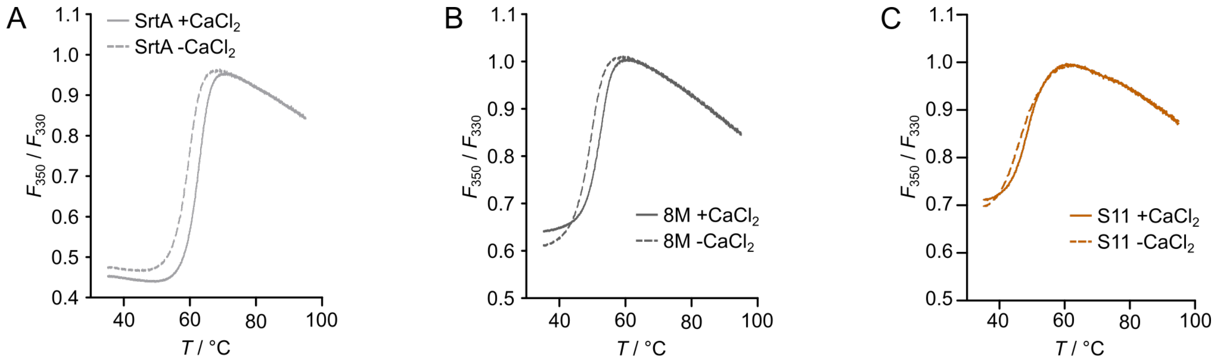

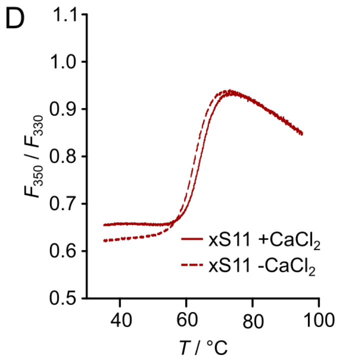

E

	T_i (-$CaCl_2$) / °C	T_i (+$CaCl_2$) / °C
SrtA	59.6	62.8
8M	49.6	52.4
S11	46.0	48.6
xS11	62.5	64.4

Figure S16: Melting curves of **SrtA**, **8M**, **S11** and **xS11** determined by nanoDSF. Protein (c = 5–10 µM) was used in a buffer with and without 5 mM $CaCl_2$ and the change in F_{350}/F_{330} ratio was monitored in the range of 30–95 °C. Melting temperatures (T_i) are an average of triplicate measurements.

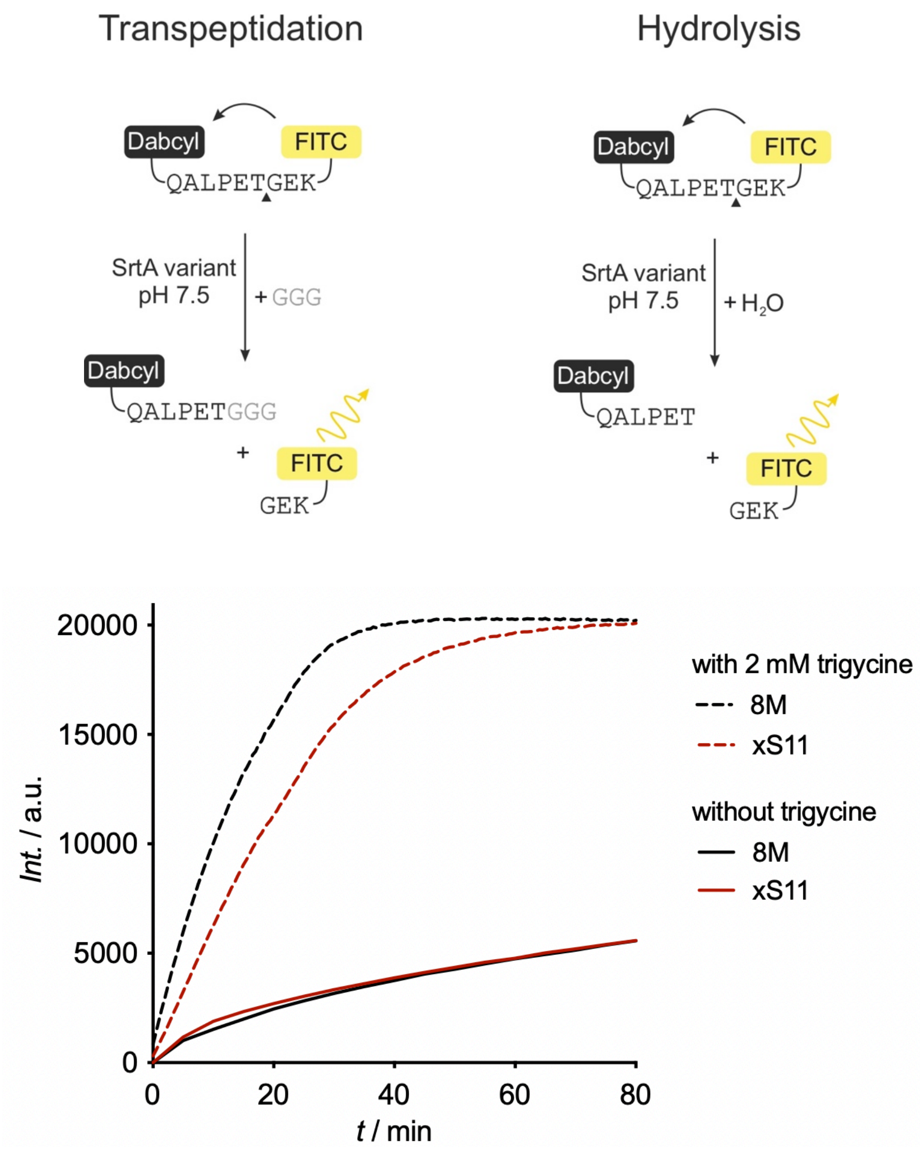

Figure S11: Top: Scheme for Sortase-mediated reaction of probe with triglycine (transpeptidation) and water (hydrolysis). Bottom: Fluorescence intensity measurement of enzymatic reaction performed in the absence (solid line) and the presence of triglycine (c = 2 mM, dashed line).

A) Peak t_r = 10.3 min: substrate (**a**)

MW(calculated) = 1612.73 Da

	m/z calculated	m/z found
$[M+2H]^{+2}$	807.37	806.6
$[M+3H]^{+3}$	538.58	538.0

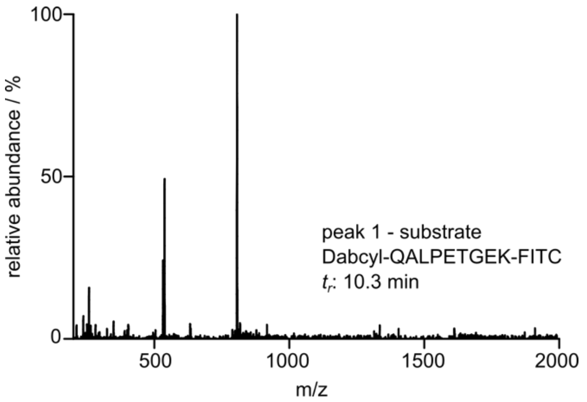

B) Peak t_r = 9.4 min: product I (**b**)

MW(calculated) = 1080.17 Da

	m/z calculated	m/z found
$[M+1H]^{+1}$	1081.17	1080.6
$[M+2H]^{+2}$	541.09	540.9

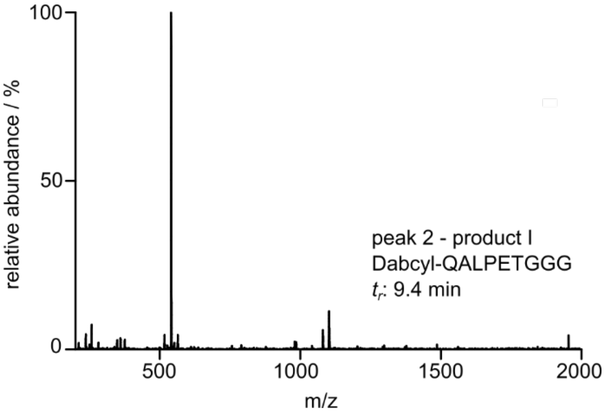

C) Peak t_r = 7.8 min: product II (**c**)

MW(calculated) = 721.74 Da

	m/z calculated	m/z found
$[M+1H]^{+1}$	722.74	721.1
$[M+2H]^{+2}$	361.87	361.2

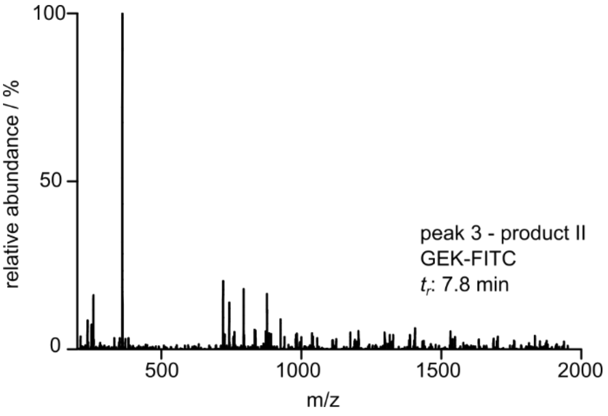

Figure S12: **xS11** transpeptidase reaction under denaturing conditions. MS spectrum of substrate (a), product I (b) and product II (c) of the labelled peptide probe analyzed by HPLC-MS including calculated and found mases.

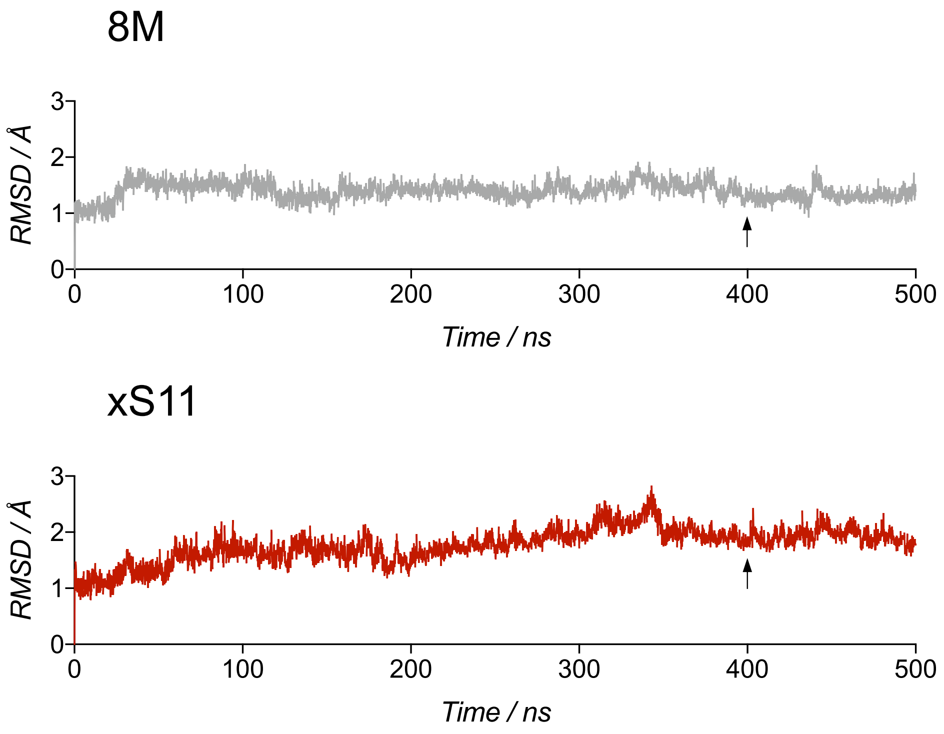

Figure S19: Sortase variants **8M** and cross-linked **xS11** were equilibrated extensively by a 500 ns molecular dynamics simulation to ensure complete minimization of the modified structures. Backbone atom (Cα+C+N) RMSD values for non-terminal residues (71–202) are displayed, excluding the flexible termini. A structural snapshot (indicated) for both 8M and xS11 was used for subsequent MD simulations and analysis (Figure 4 and Supplementary Figure 19). Snapshots coordinates are available as Supplementary Information).

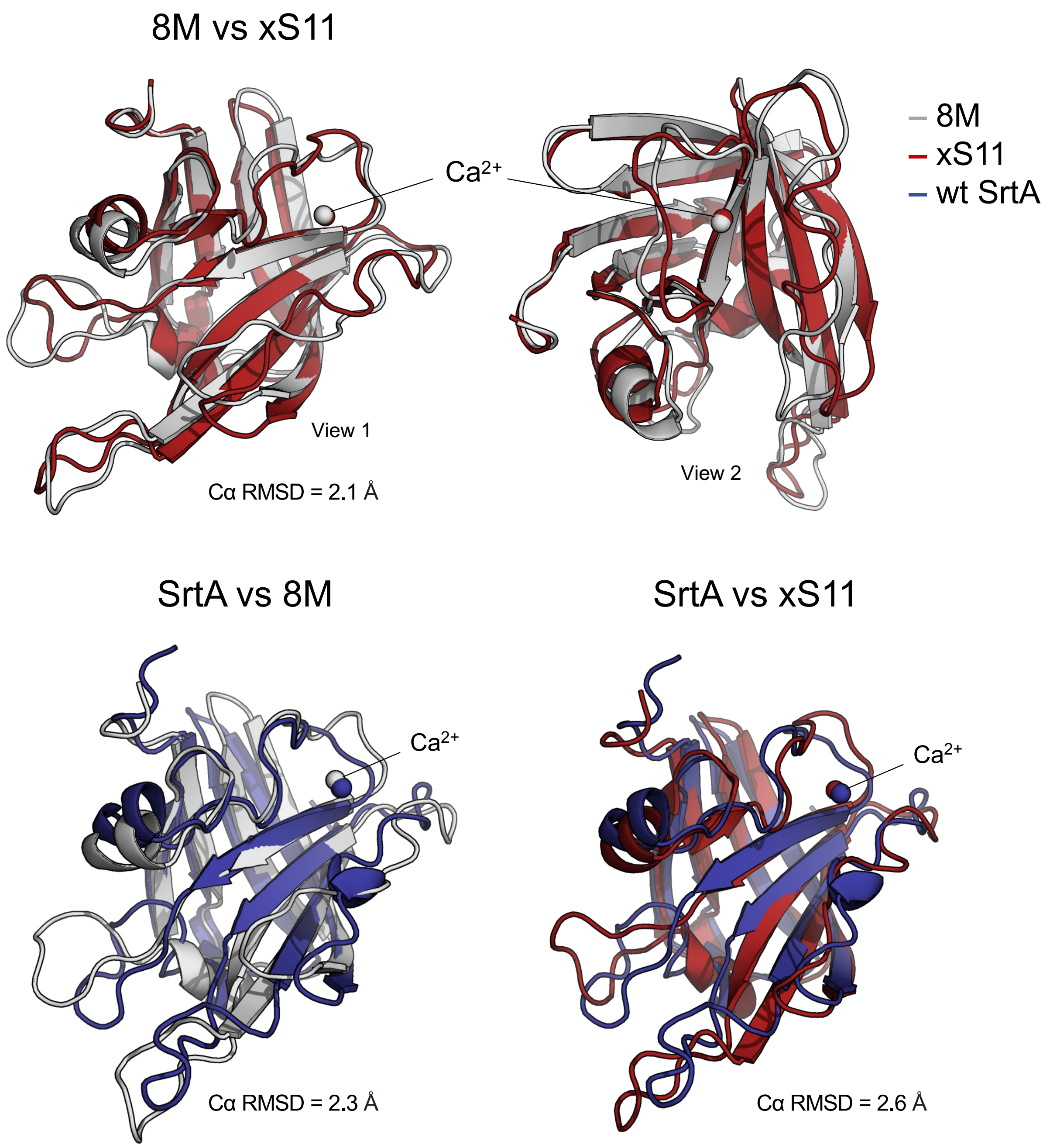

Figure S20: The tertiary structures of **8M** (grey) and **xS11** (red) do not deviate significantly relative to the NMR structure of wt SrtA (blue, PDB ID = 2KID). Equilibrated structures of **8M** and **xS11** represent the 400 ns snapshot of the above 500 ns simulation (Supplementary Figure S18). RMSD values indicate carbon-alpha deviations of residues 71–202, demonstrating some variation relative to the wild-type SrtA structure due to loop rearrangements (RMSD 2–3 Å), whilst the core secondary structure remains constant. The calcium position is nearly identical for 8M and crosslinked **xS11** - within 0.2 Å between these two snapshots.

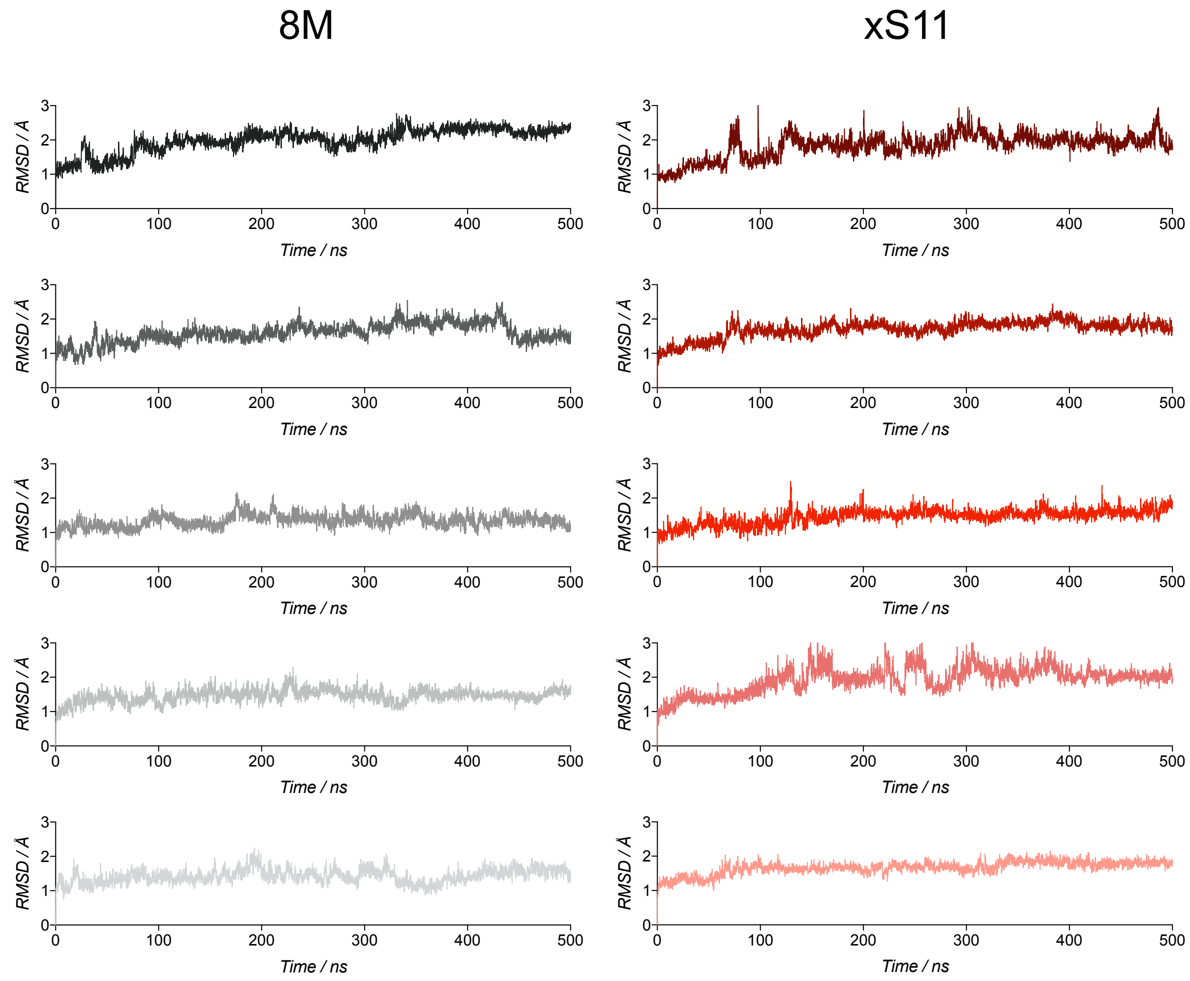

Figure S21: (A) Backbone atom (Cα, C, N) RMSD values relative to the equilibrated structures (Supplementary Figure 18) for sortase variants **8M** (left) and **xS11** (right) over the course of five replicate 500 ns molecular dynamics trajectories. RMSD values are calculated for all non-terminal residues (71–202). Consistently stable trajectories for both un-crosslinked and crosslinked variants suggested adequate equilibration of the two models within the MD forcefield, enabling analysis of protein flexibility under native conditions (Figure 4).
